# Pentoxifylline improves the quality of life in type-2 diabetes foot syndrome

**DOI:** 10.12669/pjms.35.5.11

**Published:** 2019

**Authors:** Marwan Al-Nimer, Rawa Ratha, Taha Mahwi

**Affiliations:** 1 Dr. Marwan Al-Nimer, MD, PhD. Department of Pharmacology and Toxicology, Hawler Medical University, Erbil-Iraq. University of Sulaimani, Sulaimani, Iraq; 2 Dr. Rawa Ratha. M.Sc. Department of Pharmacology and Toxicology, College of Pharmacy, University of Sulaimani, Sulaimani, Iraq; 3 Dr. Taha Mahwi, FRCP. Department of Medicine, College of Medicine, University of Sulaimani, Sulaimani, Iraq

**Keywords:** Diabetic foot syndrome, Pentoxifylline, Quality of life, Revised neuropathy disability score

## Abstract

**Objectives:**

To evaluate the effect of pentoxifylline on the quality of life (QoL) in diabetic foot syndrome (DFS) by using Short Form-36 questionnaire, and in reference to the revised neuropathy disability score (RNDS) and grading of diabetic foot.

**Methods:**

This randomized placebo-controlled study was carried in the Department of Pharmacology at University of the Sulaimani through 2018. A total number of 80 T2D patients were recruited from outpatients Department attended the Center of Diabetes and the Shar Teaching Hospital in the University of Sulaimani, Sulaimani-Iraq. Group I (non-DFS, n=40) were subgrouped into Group-IA treated with placebo (n=20), and Group-IB treated with 400 mg pentoxifylline thrice daily for 8 weeks. Group II (DFS, n=40) sub grouped into Group-IIA treated with placebo (n=20), and Group-IIB treated with pentoxifylline. The primary outcome measures including the data of SF-36, RNDS, and grading of diabetic foot.

**Results:**

Pentoxifylline therapy significantly reduced the RNDS, improved the clinical evidence of diabetic foot, improved the QoL particularly the domains that related to emotional problems and physical health. Pentoxifylline offered a better effect in DFS compared with non-DFS patients

**Conclusion:**

Pentoxifylline treatment improves the quality of life in diabetic foot syndrome and its effect is related to the scoring of revised neuropathy disability and grading of diabetic foot.

## INTRODUCTION

Type-2 diabetes mellitus (T2D) is a chronic multi-systemic disease with microvascular and/or macrovascular complications. The clinical features of diabetic foot syndrome (DFS) includes ulceration, infection, destruction of soft tissue and gangrene due to the diabetic neuropathy and/ or peripheral artery disease.^1^-^3^ Patients with T2D complain of depression because diabetes carries a high morbidity rate, and low quality of the life (that assessed by Short Form (SF)-36), with a significant high percentage among female gender.^4^ Diabetic patients with peripheral neuropathy have a significant low score of SF-36 particularly of the pain and general health domains.^5^ Pharmacological intervention is useful in the management of diabetic foot syndrome to improve the nerve function, and the peripheral circulation for preserving the initial QoL.^6^ Pentoxifylline (PXF) is a phosphodiesterase enzyme that is related to methylxanthine, improves the peripheral circulation by its rheological and vasodilatory properties.^7^,^8^ It is useful in management of the peripheral artery disease.^9^ Short term therapy of 400mg thrice daily of PXF for four weeks improves the quality of the life (QoL) in cancer cachexia patients but it deteriorates the QoL after eight weeks treatment without producing a significant effect on the body weight.^10^ The rationale of this study is to evaluate whether the effect of PXF on the DFS differs from the T2D patients without diabetic foot because DFS patients usually suffer from peripheral artery disease. PXF can improve the peripheral circulation and this may reflect on the general health of the patients. This randomized placebo-controlled study aimed to evaluate the effect of PXF on the QoL of T2D patients and DFS and T2D by using SF-36.

## METHODS

The study was conducted in the Department of Pharmacology, College of Medicine at University of the Sulaimani in cooperation with the Shar Teaching Hospital in the Sulaimani governorate-Iraq through 2018. The Institutional Scientific Committee at the University of Sulaimani approved this randomized placebo-controlled study, according to the guidelines Helsinki. Drugs or devices that indicated to the patients should not be harmful, and the patient is free to withdraw from the study. A consent form was obtained from each patient prior to the admission to the study.

This is a randomized placebo controlled study. The patients were recruited from the Shar Teaching Hospital and the Center of Diabetes from outpatient’s departments in the Sulaimani-Iraq. The eligible patients were adult males and females. The criteria of inclusion were diagnosed patients of T2D irrespective of duration of disease. The diagnosis of DFS confirmed by the consultants of Endocrinology using the Wagner-Meggitt classification of DFS.^11^ This classification is simple and provides information related to the objective of the study compared with other classifications. Wagner-Meggitt classification grading the DFS into six grades (0–5) of lesions. In this study patients with Grade 0, 1, and 2 of Wagner classification were included.

The criteria of exclusion are Type-1 diabetes, clinical evidence of complications of diabetes (including retinopathy, nephropathy, and current cardiovascular events), smoking, pregnancy and lactating and nursing mother’s chronic liver and kidney diseases, and drug intake, including steroidal and non-steroidal anti-inflammatory medicines. Randomized tables were used to achieve the randomization. Consultants of endocrinology and the authors examined each patient thoroughly.

Pentoxifylline-treated patients were used 400 mg oral dose thrice daily for eight weeks and the placebo-treated patients were taken an equivalent oral dose of methylcellulose in form of capsule (size #1) with the same schedule of pentoxifylline-treated patients. Antidiabetic drugs and in case of DFS the local dressing and antibiotic treatment were already taken by the patients. Literature review did not mention any effect of pentoxifylline or methylcellulose on the antidiabetic medicines.

### Sample Size Estimation

The sample size of two independent samples paired samples was calculated after doing the pilot study on the T2D patients with DFS and without DFS. The mean, standard deviations, and the difference between the means were calculated from the pilot study. The power of the study 1-β (where the β is type II error) is fixed at 80% (0.8) and the significance level (α), which is the type I error, is fixed at 5% (≤0.05). Then the following equations were used to calculate the sample size:

Sample size per group in unpaired data = 1 + 2C ⊆ (Standard deviation/difference between means).^2^ Sample size per group in paired data = 2 + C⊆ (Standard deviation/difference between means).^2^ Where C represents the Constant value that derived from the statistical tables and it equals to 7.85 when the 1-β =0.8 and α=0.05.

### Clinical Assessment

The authors examined and interviewed each patient taking characteristics of the patients and obtaining the following primary outcome measures: fasting serum glucose, glycated hemoglobin, subjective and objective signs and symptoms of neuropathy, and SF-36 data. Fasting serum glucose (mg/dl) and glycated hemoglobin (HbA_1c_ %) were determined in the laboratories of the Center of Diabetes as routine investigations. Diabetic peripheral neuropathy was assessed on the basis of subjective and objective symptoms (pain, numbness, vibration, tactile and temperature sensations, pin prick and ankle reflex) using the scoring of 0 = absent and 1 = reduced or present for each side of the body. Patients with a revised neuropathy disability score (RNDS) of six points or more are considered to show abnormal reaction.^12^ A consultant endocrinologist examined the neurological tests including pain sensation that assessed by pin-prick test, vibration sensation by using 128Hz tuning fork, tactile sensation by using monofilament test, thermal sensation by application of cold and warm objects, and the ankle reflex by tapping the Achilles tendon with medical hammer.

Short Form-36 is a measure of health state of patients that assessed physical functioning, role limitations due to physical health, role limitations due to emotional problems, energy / fatigue, emotional well-being, social functioning, pain, and general health. The researcher interviewed each patient and the patients had to answer each question of SF-36. Each question has a number of options with a different score. The lower total score indicates the more disability, and the higher total score indicates the less disability. Zero score indicates maximum disability, and a score of 100 indicates that there is no disability.^13^

A total number 80 patients who fulfilled the above criteria were included in the study. The patients were grouped into: Group-I (n=40): T2D without clinical evidence of diabetic foot. This group was divided equally (n=20, each) into Group-IA received placebo treatment in the form of methylcellulose + anti diabetic drugs, and Group-IB treated with PXF 400mg thrice daily for 8 weeks + anti diabetic drugs.

Group-II (n=40): Patients with DFS and this group divided equally (n=20, each) into Group IIA received placebo treatment in the form of methylcellulose + anti diabetic drugs + traditional treatment of diabetic foot (antibiotics + dressings), and Group-IIB treated with PXF 400mg thrice daily for 8 weeks + anti diabetic Drugs + traditional treatment of diabetic foot (antibiotics + dressings). A baseline data of RNDS and SF-36 were determined before treatment and after 8 week of treatment.

### Statistical Analysis

The results were provided in number, percentage and as mean ± SD. Independent two samples and paired student’s t test (two-tailed) were used to compare the mean between groups. Chi-square test was used for dichotomous data. The differences were considered statistically significant when p <0.05. All calculations were made using Excel 2003 (Microsoft Corporation, Redmond, WA, USA) and IBM-Statistical Package for the Social Sciences statistics 20 software (USA).

## RESULTS

**Fig.1:**
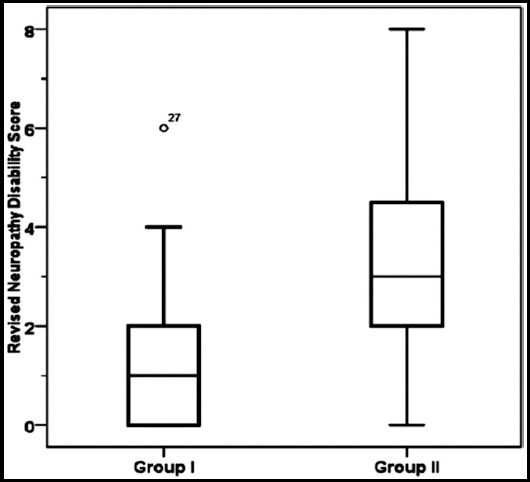
Group II patients have a significant (P<0.001) high revised neuropathy disability score compared with Group-I. Group-I: non-diabetic foot syndrome, Group-II: diabetic foot syndrome.

A total number of 80 patients were included in this study. The age, duration of diabetes, and the fasting serum glucose were significantly higher than the corresponding values in the Group-I. Table-I Clinical evidence of previous diabetic foot represented by amputation was accounted 12.5% of Group-II patients. The distribution of Group-II patients according to the Wagner- Meggitt classification were 12.5%, 52.5% and 35% for grade 0, 1, and 2 respectively. Group-II patients have significant low means ± SDs of the eight domains of SF=36 compared with Group-I, and the domains of role of limitation due to emotional problems and physical health comprised high percentages of deterioration in reference to Group-I (Table-II). Fig.1 shows that the mean ± SD of RNDS was significantly (p<0.001) higher in Group-II (3.35 ±2.11) compared with Group-I (1.35±1.48). Group-I patients treated with 400mg thrice daily for 8week showed improvement of the eight domains of SF-36 compared with placebo treatment (Table-III). Pentoxifylline improves the role of limitation due to emotional problems and physical health by 55% and 35% increment, respectively. Group-II patients treated with 400mg thrice daily for 8week showed improvement of the eight domains of SF-36 compared with placebo treatment (Table-IV). Pentoxifylline improves the role of limitation due to emotional problems and physical health by 60% and 40% increment, respectively.

**Table I:** Characteristics of the patients enrolled in this study.

Variables	Group-I (n=40)	Group-II (n=40)	P-value
Age	51.7±10.2	56.0±8.0	0.041
Gender (Female: Male)	23:17	28:12	0.657
Duration of diabetes (year)	7.3±4.5	10.5±6.1	0.008
Family history of diabetes	23	24	0.820
***Clinical features***			
Loss of leg sensation	20	26	0.174
Calf muscle pain	24	30	0.152
Amputation	0	5	0.021
***Wagner grading score***			
0	0	5	
1	0	21	<0.001
2	0	14	
Fasting serum glucose (mg/dl)	198.7±67.0	231.9±79.8	0.047
Glycated hemoglobin (%)	9.15±1.72	9.53±2.06	0.370

The results are expressed as number and mean ± SD. P-value calculated by independent two samples for continuous data and Chi square test for categorized data. Group-I: non-diabetic foot syndrome, Group-II: diabetic foot syndrome.

**Table II:** Assessment of the quality of life of non-diabetic foot syndrome and diabetic foot syndrome patients assessed by Short Form Health scale (SF-36).

Variables	Group-I (n=40)	Group-II (n=40)	Percentages changes	P-value
Physical functioning	53.8±25.7	28.4±20.6	47.2$	<0.001
Role limitations due to physical health	11.9±30.5	0.63±4.0	94.7$	0.023
Role limitations due to emotional problems	15.0±33.7	0.0±0.0	100$	0.006
Energy/Fatigue	37.8±15.3	17.3±11.3	54.2$	<0.001
Emotional well being	44.9±11.1	37.9±9.9	15.6$	0.004
Social functioning	59.1±20.8	27.5±20.1	53.5$	<0.001
Pain	60.8±17.8	39.1±15.8	35.7$	<0.001
General health	35.4±15.8	11.8±11.4	66.7$	<0.001

The results are expressed as mean ± SD. P-value calculated by using independent two samples, Two tailed t- test. Group I: non-diabetic foot syndrome, Group II: diabetic foot syndrome.

**Table III:** Effect of placebo and pentoxifylline (400mg thrice daily, for 8 weeks) treatment on the quality of life of diabetic patients without diabetic foot syndrome assessed by Short Form Health scale (SF-36).

	Group IA (n=20)				Group IB (n=20)			

Domains	Before treatment	After treatment	Scoring changes	p-value	Before treatment	After treatment	Scoring changes	p-value
Physical functioning	46.8±25.2	46.0±26.2	-0.8	0.506	60.8±24.8	73.0±22.3	+12.2	<0.001
Role limitations due to physical health	3.8±9.2	16.3±16.8	+12.5	0.014	20.0±41.0	55.0±31.0	+35.0	<0.001
Role limitations due to emotional problems	18.3±36.6	23.3±21.9	+5.0	0.508	11.7±31.1	66.7±32.4	+55.0	<0.001
Energy/Fatigue	36.3±17.8	32.0±15.5	-4.3	0.440	39.3±12.5	48.8±8.4	+9.5	<0.001
Emotional well being	45.4±12.1	39.4±11.1	-6.0	0.171	44.4±10.4	51.4±8.4	+7.0	<0.001
Social functioning	55.0±21.2	48.1±23.4	-6.9	0.260	63.1±20.1	71.9±15.1	+8.8	0.003
Pain	61.0±17.3	52.8±19.7	-8.2	0.122	60.5±18.6	74.8±15.3	+14.3	<0.001
General health	31.5±14.5	26.5±16.3	-5.0	0.359	39.2±16.5	56.2±12.0	+17.0	<0.001

The results are expressed as mean ± SD. P-value is calculated by using paired two tailed student t- test (n=20. each). Group-IA: placebo pretreatment, Group-IB: pentoxifylline pretreatment.

**Table IV:** Effect of placebo and pentoxifylline (400mg thrice daily, for 8 weeks) treatment on the quality of life of diabetic patient’s diabetic foot syndrome assessed by Short Form Health scale (SF-36).

Domains	Group IIA				Group IIB			

	Before treatment	After treatment	Scoring changes	p-value	Before treatment	After treatment	Scoring changes	p-value
Physical functioning	21.5±19.5	17.8±17.4	-3.7	0.005	35.3±19.8	49.8±22.8	+14.5	<0.001
Role limitations due to physical health	1.3±5.6	1.3±5.6	0	1.000	0.0±0.0	40.0±29.7	+40.0	<0.001
Role limitations due to emotional problems	0±0	1.7±7.5	+1.7	0.330	0.0±0.0	60.0±33.5	+60.0	<0.001
Energy/Fatigue	15.3±11.8	12.5±11.2	-2.8	0.102	19.3±10.4	32.3±9.5	+13.0	<0.001
Emotional well being	37.4±9.5	32.2±1.5	-5.2	0.008	38.4±10.6	45.4±9.6	+7.0	<0.001
Social functioning	23.8±21.8	16.3±16.3	-7.5	0.004	31.3±17.9	38.8±19.0	+7.5	0.007
Pain	34.3±16.9	32.0±19.2	-2.3	0.420	44.0±13.3	54.8±16.2	+10.8	0.006
General health	10.5±12.4	4.8±9.	-5.7	0.001	13.0±10.4	32.0±14.4	+19.0	<0.001

The results are expressed as mean ± SD. P value is calculated by using paired two tailed student t- test (n=20. each). Group IIA: placebo pretreatment, Group IIB: pentoxifylline treatment.

The effects of PXF against the domains of SF-36 in in non-DFS (Group-I) and DFS (Group-II) were comparable. In Group-I, placebo did not induce significant changes in the RNDS which increased from 1.45±1.61 to 2.0±0.94, p=0.066, while PXF produced a significant decrease of RNDS from 1.25±1.37 to 0.25±0.91, p=0.004. In Group II, placebo did not induce significant changes in the RNDS which increased from 3.7±2.45 to 4.0±1.92, p=0.494, while PXF produced a significant decrease of RNDS from 3.0±1.69 to 1.75±2.2, p=0.001. Clinical evidence of improvement of diabetic foot was observed in 15 out of 20 patients (75%) in the Group IIB, and 4 out of 20 (25%) in the Group-IIA.

## DISCUSSION

The results of this study show that PXF improves the QoL in T2D with DFS or without DFS patients, and this effect is accompanied by improvement of the neuropathy disability score. The characteristic features of DFS patients in this study, compared with non-DFS patients, are no gender predilection, significant increase of age, long duration of diabetes, high fasting serum glucose with clinical evidence of diabetic foot. This observation confirmed previous studies.^14^,^15^ A significant high RNDS that observed in the Group II confirmed that one of the causes of DFS is peripheral neuropathy.^16^ Patients with DFS are prone to the emotional problems including a fear from infected foot or amputation, and this explains a higher percentage of impairment of the domain of role of limitation due to emotional problems.^17^ Hoban et al found that the scores of SF-36 of the DFS patients did not significantly differ from those non-DFS who complained of problems in the mental health including anxiety and depression.^18^ Simson et al reported an impairment of SF-12 score accompanied with depression and anxiety.^19^ Group II patients complained of structural changes in their foot including claw toes, equine ankle and others which reflected on the impairment of the physical health.^20^ It is well known that PXF improves the microcirculation by decreasing the red cell deformability and this study adds new information that PXF significantly reduced the RNDS. Our observation confirmed an early study that reported a favorable effect of PXF against impairment of vibration in T2D.^21^ Other studies documented that PXF reduces the burning in patients with oral mucosa fibrosis.^22^ Therefore, PXF through its effects against the peripheral sensation, which is impaired in T2D, can eliminate the role of peripheral neuropathy and thereby improves the grading of Wagner- Meggitt classification of diabetic foot; 75% of patients improved with PXF while 25% patients improved with placebo which is expected as the placebo treatment may show a beneficial effect in 30% in the clinical studies. Moreover, PXF improves the QoL in non-DFS and DFS patients equally suggesting that PXF exerts two important effects in diabetes; the first is: it improves the microcirculation and the second is: it preserves the peripheral sensation.

### Limitations of the study

It is the small sample size because it is a single center study and inclusion of diabetics irrespective of disease duration etc.

## CONCLUSION

We conclude that pentoxifylline improves the quality of the life in type-2 diabetes without foot complication or with diabetic foot.
